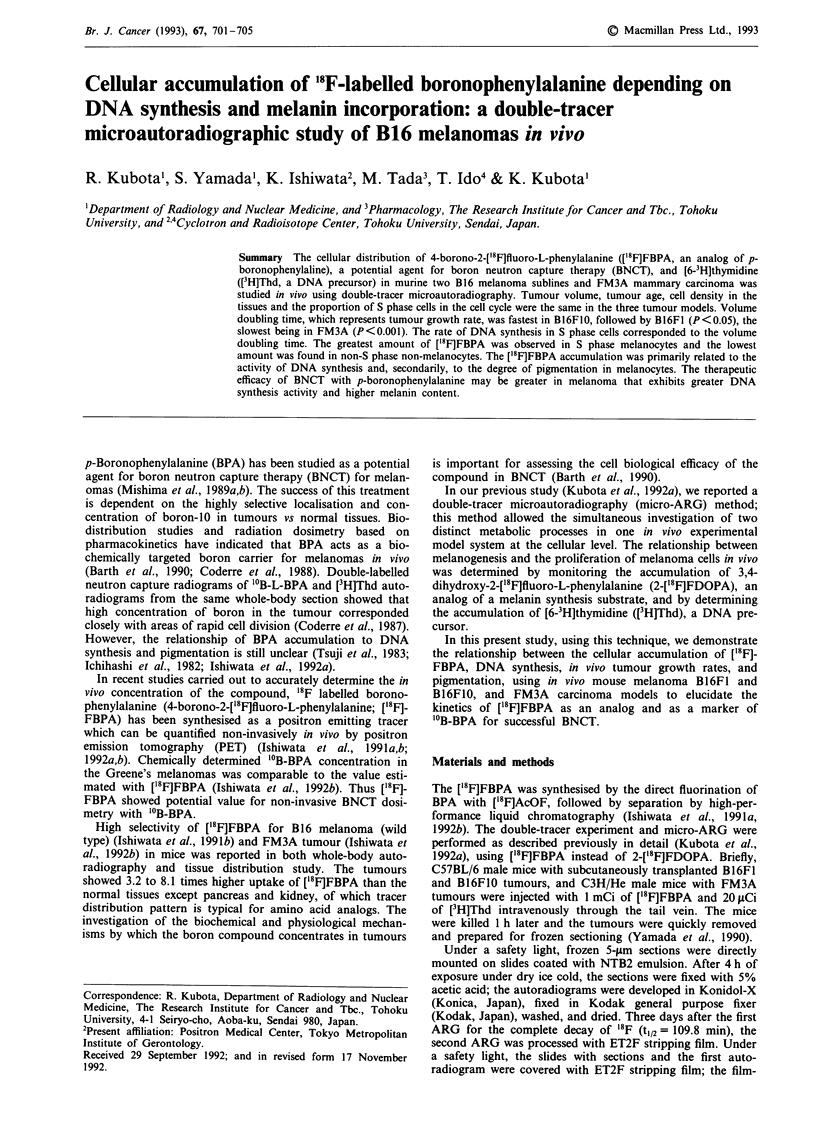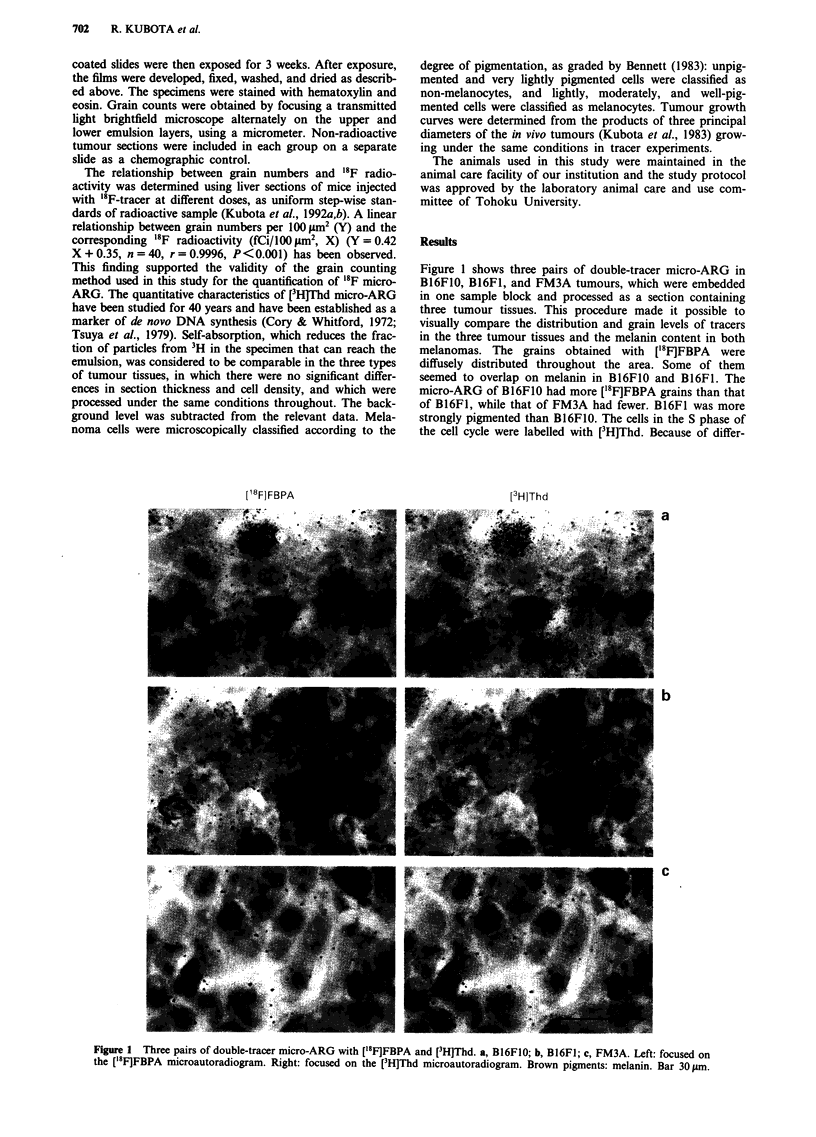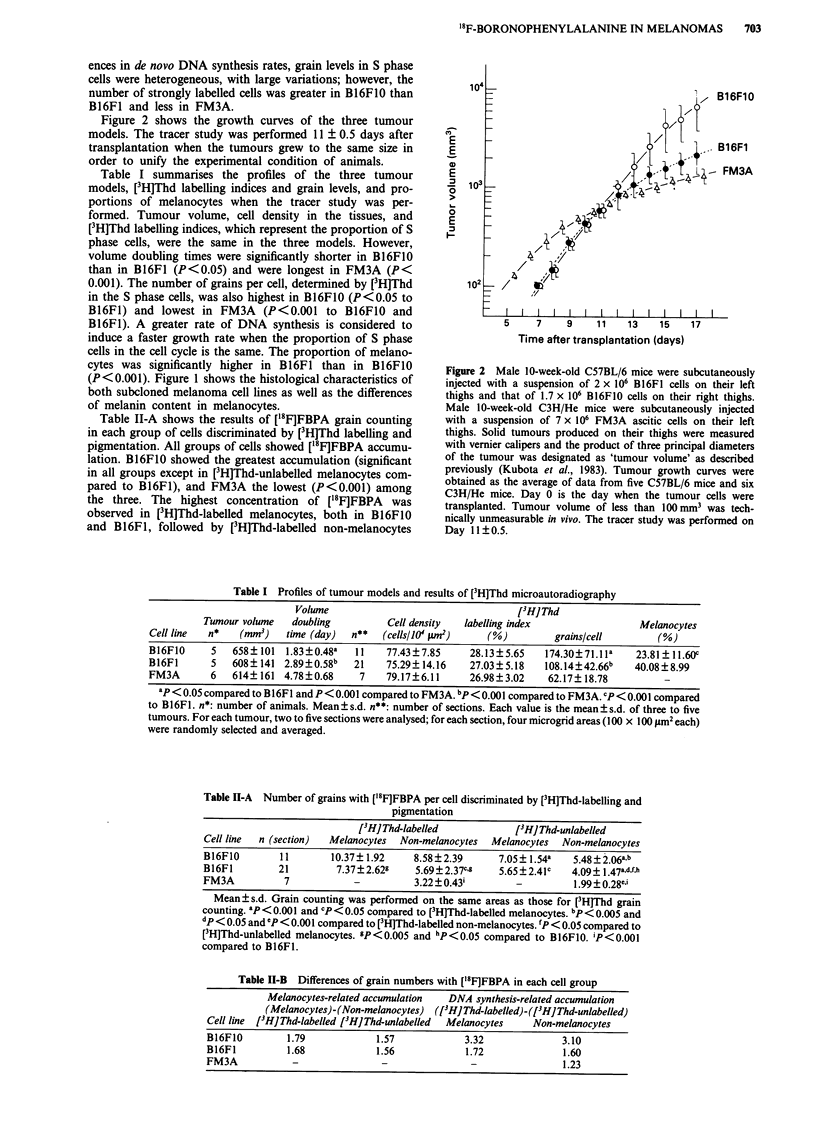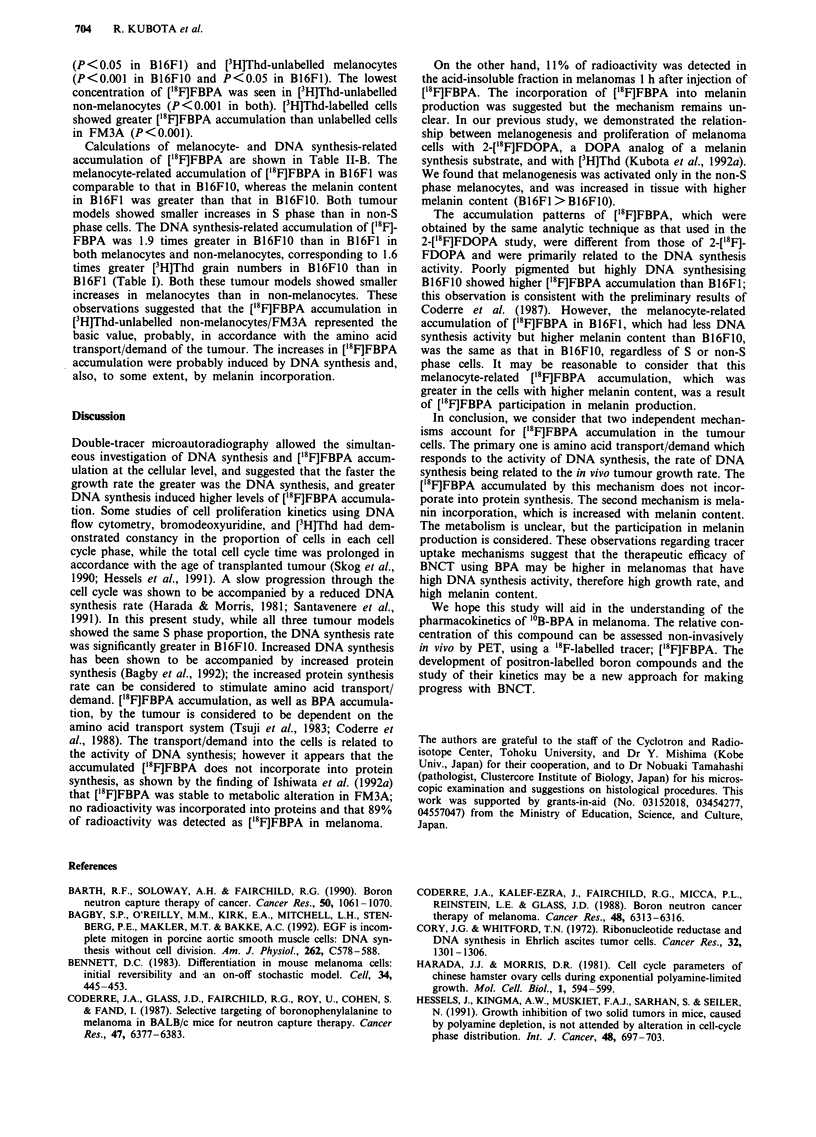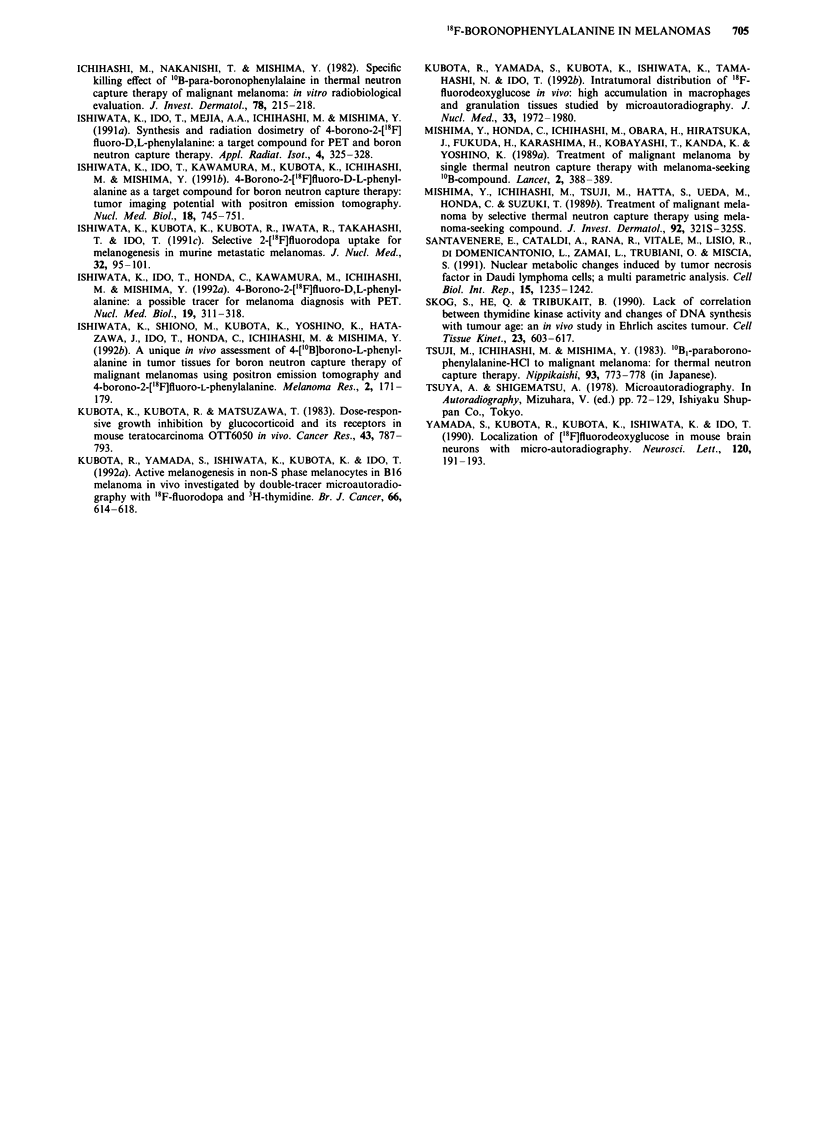# Cellular accumulation of 18F-labelled boronophenylalanine depending on DNA synthesis and melanin incorporation: a double-tracer microautoradiographic study of B16 melanomas in vivo.

**DOI:** 10.1038/bjc.1993.129

**Published:** 1993-04

**Authors:** R. Kubota, S. Yamada, K. Ishiwata, M. Tada, T. Ido, K. Kubota

**Affiliations:** Department of Radiology and Nuclear Medicine, Tohoku University, Sendai, Japan.

## Abstract

**Images:**


Br. J. Cancer (1993), 67, 701–705 © Macmillan Press Ltd., 1993

### Cellular accumulation of $^{18}$F-labelled boronophenylalanine depending on DNA synthesis and melanin incorporation: a double-tracer microautoradiographic study of B16 melanomas *in vivo*

R. Kubota[1], S. Yamada[1], K. Ishiwata[2], M. Tada[3], T. Ido[4] & K. Kubota[1]

[1]*Department of Radiology and Nuclear Medicine, and* [3]*Pharmacology, The Research Institute for Cancer and Tbc., Tohoku University, and* [2,4]*Cyclotron and Radioisotope Center, Tohoku University, Sendai, Japan.*

**Summary** The cellular distribution of 4-borono-2-[$^{18}$F]fluoro-L-phenylalanine ([$^{18}$F]FBPA, an analog of *p*-boronophenylaline), a potential agent for boron neutron capture therapy (BNCT), and [6-$^{3}$H]thymidine ([$^{3}$H]Thd, a DNA precursor) in murine two B16 melanoma sublines and FM3A mammary carcinoma was studied *in vivo* using double-tracer microautoradiography. Tumour volume, tumour age, cell density in the tissues and the proportion of S phase cells in the cell cycle were the same in the three tumour models. Volume doubling time, which represents tumour growth rate, was fastest in B16F10, followed by B16F1 ($P<0.05$), the slowest being in FM3A ($P<0.001$). The rate of DNA synthesis in S phase cells corresponded to the volume doubling time. The greatest amount of [$^{18}$F]FBPA was observed in S phase melanocytes and the lowest amount was found in non-S phase non-melanocytes. The [$^{18}$F]FBPA accumulation was primarily related to the activity of DNA synthesis and, secondarily, to the degree of pigmentation in melanocytes. The therapeutic efficacy of BNCT with *p*-boronophenylalanine may be greater in melanoma that exhibits greater DNA synthesis activity and higher melanin content.

*p*-Boronophenylalanine (BPA) has been studied as a potential agent for boron neutron capture therapy (BNCT) for melanomas (Mishima *et al.*, 1989*a,b*). The success of this treatment is dependent on the highly selective localisation and concentration of boron-10 in tumours *vs* normal tissues. Biodistribution studies and radiation dosimetry based on pharmacokinetics have indicated that BPA acts as a biochemically targeted boron carrier for melanomas *in vivo* (Barth *et al.*, 1990; Coderre *et al.*, 1988). Double-labelled neutron capture radiograms of $^{10}$B-L-BPA and [$^{3}$H]Thd autoradiograms from the same whole-body section showed that high concentration of boron in the tumour corresponded closely with areas of rapid cell division (Coderre *et al.*, 1987). However, the relationship of BPA accumulation to DNA synthesis and pigmentation is still unclear (Tsuji *et al.*, 1983; Ichihashi *et al.*, 1982; Ishiwata *et al.*, 1992*a*).

In recent studies carried out to accurately determine the *in vivo* concentration of the compound, $^{18}$F labelled boronophenylalanine (4-borono-2-[$^{18}$F]fluoro-L-phenylalanine; [$^{18}$F]-FBPA) has been synthesised as a positron emitting tracer which can be quantified non-invasively *in vivo* by positron emission tomography (PET) (Ishiwata *et al.*, 1991*a,b*; 1992*a,b*). Chemically determined $^{10}$B-BPA concentration in the Greene's melanomas was comparable to the value estimated with [$^{18}$F]FBPA (Ishiwata *et al.*, 1992*b*). Thus [$^{18}$F]-FBPA showed potential value for non-invasive BNCT dosimetry with $^{10}$B-BPA.

High selectivity of [$^{18}$F]FBPA for B16 melanoma (wild type) (Ishiwata *et al.*, 1991*b*) and FM3A tumour (Ishiwata *et al.*, 1992*b*) in mice was reported in both whole-body autoradiography and tissue distribution study. The tumours showed 3.2 to 8.1 times higher uptake of [$^{18}$F]FBPA than the normal tissues except pancreas and kidney, of which tracer distribution pattern is typical for amino acid analogs. The investigation of the biochemical and physiological mechanisms by which the boron compound concentrates in tumours is important for assessing the cell biological efficacy of the compound in BNCT (Barth *et al.*, 1990).

In our previous study (Kubota *et al.*, 1992*a*), we reported a double-tracer microautoradiography (micro-ARG) method; this method allowed the simultaneous investigation of two distinct metabolic processes in one *in vivo* experimental model system at the cellular level. The relationship between melanogenesis and the proliferation of melanoma cells *in vivo* was determined by monitoring the accumulation of 3,4-dihydroxy-2-[$^{18}$F]fluoro-L-phenylalanine (2-[$^{18}$F]FDOPA), an analog of a melanin synthesis substrate, and by determining the accumulation of [6-$^{3}$H]thymidine ([$^{3}$H]Thd), a DNA precursor.

In this present study, using this technique, we demonstrate the relationship between the cellular accumulation of [$^{18}$F]-FBPA, DNA synthesis, *in vivo* tumour growth rates, and pigmentation, using *in vivo* mouse melanoma B16F1 and B16F10, and FM3A carcinoma models to elucidate the kinetics of [$^{18}$F]FBPA as an analog and as a marker of $^{10}$B-BPA for successful BNCT.

### Materials and methods

The [$^{18}$F]FBPA was synthesised by the direct fluorination of BPA with [$^{18}$F]AcOF, followed by separation by high-performance liquid chromatography (Ishiwata *et al.*, 1991*a*, 1992*b*). The double-tracer experiment and micro-ARG were performed as described previously in detail (Kubota *et al.*, 1992*a*), using [$^{18}$F]FBPA instead of 2-[$^{18}$F]FDOPA. Briefly, C57BL/6 male mice with subcutaneously transplanted B16F1 and B16F10 tumours, and C3H/He male mice with FM3A tumours were injected with 1 mCi of [$^{18}$F]FBPA and 20 $\mu$Ci of [$^{3}$H]Thd intravenously through the tail vein. The mice were killed 1 h later and the tumours were quickly removed and prepared for frozen sectioning (Yamada *et al.*, 1990).

Under a safety light, frozen 5-$\mu$m sections were directly mounted on slides coated with NTB2 emulsion. After 4 h of exposure under dry ice cold, the sections were fixed with 5% acetic acid; the autoradiograms were developed in Konidol-X (Konica, Japan), fixed in Kodak general purpose fixer (Kodak, Japan), washed, and dried. Three days after the first ARG for the complete decay of $^{18}$F ($t_{1/2} = 109.8$ min), the second ARG was processed with ET2F stripping film. Under a safety light, the slides with sections and the first autoradiogram were covered with ET2F stripping film; the film-

Correspondence: R. Kubota, Department of Radiology and Nuclear Medicine, The Research Institute for Cancer and Tbc., Tohoku University, 4-1 Seiryo-cho, Aoba-ku, Sendai 980, Japan.
[2]Present affiliation: Positron Medical Center, Tokyo Metropolitan Institute of Gerontology.
Received 29 September 1992; and in revised form 17 November 1992.

coated slides were then exposed for 3 weeks. After exposure, the films were developed, fixed, washed, and dried as described above. The specimens were stained with hematoxylin and eosin. Grain counts were obtained by focusing a transmitted light brightfield microscope alternately on the upper and lower emulsion layers, using a micrometer. Non-radioactive tumour sections were included in each group on a separate slide as a chemographic control.

The relationship between grain numbers and $^{18}F$ radioactivity was determined using liver sections of mice injected with $^{18}F$-tracer at different doses, as uniform step-wise standards of radioactive sample (Kubota *et al.*, 1992*a*,*b*). A linear relationship between grain numbers per 100 $\mu m^2$ (Y) and the corresponding $^{18}F$ radioactivity (fCi/100 $\mu m^2$, X) ($Y = 0.42\ X + 0.35$, $n = 40$, $r = 0.9996$, $P < 0.001$) has been observed. This finding supported the validity of the grain counting method used in this study for the quantification of $^{18}F$ micro-ARG. The quantitative characteristics of [$^3H$]Thd micro-ARG have been studied for 40 years and have been established as a marker of *de novo* DNA synthesis (Cory & Whitford, 1972; Tsuya *et al.*, 1979). Self-absorption, which reduces the fraction of particles from $^3H$ in the specimen that can reach the emulsion, was considered to be comparable in the three types of tumour tissues, in which there were no significant differences in section thickness and cell density, and which were processed under the same conditions throughout. The background level was subtracted from the relevant data. Melanoma cells were microscopically classified according to the degree of pigmentation, as graded by Bennett (1983): unpigmented and very lightly pigmented cells were classified as non-melanocytes, and lightly, moderately, and well-pigmented cells were classified as melanocytes. Tumour growth curves were determined from the products of three principal diameters of the *in vivo* tumours (Kubota *et al.*, 1983) growing under the same conditions in tracer experiments.

The animals used in this study were maintained in the animal care facility of our institution and the study protocol was approved by the laboratory animal care and use committee of Tohoku University.

### Results

Figure 1 shows three pairs of double-tracer micro-ARG in B16F10, B16F1, and FM3A tumours, which were embedded in one sample block and processed as a section containing three tumour tissues. This procedure made it possible to visually compare the distribution and grain levels of tracers in the three tumour tissues and the melanin content in both melanomas. The grains obtained with [$^{18}F$]FBPA were diffusely distributed throughout the area. Some of them seemed to overlap on melanin in B16F10 and B16F1. The micro-ARG of B16F10 had more [$^{18}F$]FBPA grains than that of B16F1, while that of FM3A had fewer. B16F1 was more strongly pigmented than B16F10. The cells in the S phase of the cell cycle were labelled with [$^3H$]Thd. Because of differ-

**Figure 1** Three pairs of double-tracer micro-ARG with [$^{18}F$]FBPA and [$^3H$]Thd. **a**, B16F10; **b**, B16F1; **c**, FM3A. Left: focused on the [$^{18}F$]FBPA microautoradiogram. Right: focused on the [$^3H$]Thd microautoradiogram. Brown pigments: melanin. Bar 30 $\mu m$.

ences in *de novo* DNA synthesis rates, grain levels in S phase cells were heterogeneous, with large variations; however, the number of strongly labelled cells was greater in B16F10 than B16F1 and less in FM3A.

Figure 2 shows the growth curves of the three tumour models. The tracer study was performed 11 ± 0.5 days after transplantation when the tumours grew to the same size in order to unify the experimental condition of animals.

Table I summarises the profiles of the three tumour models, [$^3$H]Thd labelling indices and grain levels, and proportions of melanocytes when the tracer study was performed. Tumour volume, cell density in the tissues, and [$^3$H]Thd labelling indices, which represent the proportion of S phase cells, were the same in the three models. However, volume doubling times were significantly shorter in B16F10 than in B16F1 ($P<0.05$) and were longest in FM3A ($P<0.001$). The number of grains per cell, determined by [$^3$H]Thd in the S phase cells, was also highest in B16F10 ($P<0.05$ to B16F1) and lowest in FM3A ($P<0.001$ to B16F10 and B16F1). A greater rate of DNA synthesis is considered to induce a faster growth rate when the proportion of S phase cells in the cell cycle is the same. The proportion of melanocytes was significantly higher in B16F1 than in B16F10 ($P<0.001$). Figure 1 shows the histological characteristics of both subcloned melanoma cell lines as well as the differences of melanin content in melanocytes.

Table II-A shows the results of [$^{18}$F]FBPA grain counting in each group of cells discriminated by [$^3$H]Thd labelling and pigmentation. All groups of cells showed [$^{18}$F]FBPA accumulation. B16F10 showed the greatest accumulation (significant in all groups except in [$^3$H]Thd-unlabelled melanocytes compared to B16F1), and FM3A the lowest ($P<0.001$) among the three. The highest concentration of [$^{18}$F]FBPA was observed in [$^3$H]Thd-labelled melanocytes, both in B16F10 and B16F1, followed by [$^3$H]Thd-labelled non-melanocytes

**Figure 2** Male 10-week-old C57BL/6 mice were subcutaneously injected with a suspension of $2 \times 10^6$ B16F1 cells on their left thighs and that of $1.7 \times 10^6$ B16F10 cells on their right thighs. Male 10-week-old C3H/He mice were subcutaneously injected with a suspension of $7 \times 10^6$ FM3A ascitic cells on their left thighs. Solid tumours produced on their thighs were measured with vernier calipers and the product of three principal diameters of the tumour was designated as 'tumour volume' as described previously (Kubota *et al.*, 1983). Tumour growth curves were obtained as the average of data from five C57BL/6 mice and six C3H/He mice. Day 0 is the day when the tumour cells were transplanted. Tumour volume of less than 100 mm$^3$ was technically unmeasurable *in vivo*. The tracer study was performed on Day 11±0.5.

**Table I** Profiles of tumour models and results of [$^3$H]Thd microautoradiography

| *Cell line* | *Tumour volume* *n** | *Tumour volume (mm³)* | *Volume doubling time (day)* | *n*** | *Cell density (cells/10⁴ μm²)* | *[³H]Thd labelling index (%)* | *[³H]Thd grains/cell* | *Melanocytes (%)* |
|---|---|---|---|---|---|---|---|---|
| B16F10 | 5 | 658±101 | 1.83±0.48$^a$ | 11 | 77.43±7.85 | 28.13±5.65 | 174.30±71.11$^a$ | 23.81±11.60$^c$ |
| B16F1 | 5 | 608±141 | 2.89±0.58$^b$ | 21 | 75.29±14.16 | 27.03±5.18 | 108.14±42.66$^b$ | 40.08±8.99 |
| FM3A | 6 | 614±161 | 4.78±0.68 | 7 | 79.17±6.11 | 26.98±3.02 | 62.17±18.78 | – |

$^a P<0.05$ compared to B16F1 and $P<0.001$ compared to FM3A. $^b P<0.001$ compared to FM3A. $^c P<0.001$ compared to B16F1. *n**: number of animals. Mean±s.d. *n***: number of sections. Each value is the mean±s.d. of three to five tumours. For each tumour, two to five sections were analysed; for each section, four microgrid areas (100 × 100 μm$^2$ each) were randomly selected and averaged.

**Table II-A** Number of grains with [$^{18}$F]FBPA per cell discriminated by [$^3$H]Thd-labelling and pigmentation

| *Cell line* | *n (section)* | *[³H]Thd-labelled Melanocytes* | *[³H]Thd-labelled Non-melanocytes* | *[³H]Thd-unlabelled Melanocytes* | *[³H]Thd-unlabelled Non-melanocytes* |
|---|---|---|---|---|---|
| B16F10 | 11 | 10.37±1.92 | 8.58±2.39 | 7.05±1.54$^a$ | 5.48±2.06$^{a,b}$ |
| B16F1 | 21 | 7.37±2.62$^g$ | 5.69±2.37$^{c,g}$ | 5.65±2.41$^c$ | 4.09±1.47$^{a,d,f,h}$ |
| FM3A | 7 | – | 3.22±0.43$^i$ | – | 1.99±0.28$^{e,i}$ |

Mean±s.d. Grain counting was performed on the same areas as those for [$^3$H]Thd grain counting. $^a P<0.001$ and $^c P<0.05$ compared to [$^3$H]Thd-labelled melanocytes. $^b P<0.005$ and $^d P<0.05$ and $^e P<0.001$ compared to [$^3$H]Thd-labelled non-melanocytes. $^f P<0.05$ compared to [$^3$H]Thd-unlabelled melanocytes. $^g P<0.005$ and $^h P<0.05$ compared to B16F10. $^i P<0.001$ compared to B16F1.

**Table II-B** Differences of grain numbers with [$^{18}$F]FBPA in each cell group

| *Cell line* | *Melanocytes-related accumulation (Melanocytes)-(Non-melanocytes)* [³H]Thd-labelled | *Melanocytes-related accumulation (Melanocytes)-(Non-melanocytes)* [³H]Thd-unlabelled | *DNA synthesis-related accumulation ([³H]Thd-labelled)-([³H]Thd-unlabelled)* Melanocytes | *DNA synthesis-related accumulation ([³H]Thd-labelled)-([³H]Thd-unlabelled)* Non-melanocytes |
|---|---|---|---|---|
| B16F10 | 1.79 | 1.57 | 3.32 | 3.10 |
| B16F1 | 1.68 | 1.56 | 1.72 | 1.60 |
| FM3A | – | – | – | 1.23 |

($P<0.05$ in B16F1) and [$^3$H]Thd-unlabelled melanocytes ($P<0.001$ in B16F10 and $P<0.05$ in B16F1). The lowest concentration of [$^{18}$F]FBPA was seen in [$^3$H]Thd-unlabelled non-melanocytes ($P<0.001$ in both). [$^3$H]Thd-labelled cells showed greater [$^{18}$F]FBPA accumulation than unlabelled cells in FM3A ($P<0.001$).

Calculations of melanocyte- and DNA synthesis-related accumulation of [$^{18}$F]FBPA are shown in Table II-B. The melanocyte-related accumulation of [$^{18}$F]FBPA in B16F1 was comparable to that in B16F10, whereas the melanin content in B16F1 was greater than that in B16F10. Both tumour models showed smaller increases in S phase than in non-S phase cells. The DNA synthesis-related accumulation of [$^{18}$F]-FBPA was 1.9 times greater in B16F10 than in B16F1 in both melanocytes and non-melanocytes, corresponding to 1.6 times greater [$^3$H]Thd grain numbers in B16F10 than in B16F1 (Table I). Both these tumour models showed smaller increases in melanocytes than in non-melanocytes. These observations suggested that the [$^{18}$F]FBPA accumulation in [$^3$H]Thd-unlabelled non-melanocytes/FM3A represented the basic value, probably, in accordance with the amino acid transport/demand of the tumour. The increases in [$^{18}$F]FBPA accumulation were probably induced by DNA synthesis and, also, to some extent, by melanin incorporation.

### Discussion

Double-tracer microautoradiography allowed the simultaneous investigation of DNA synthesis and [$^{18}$F]FBPA accumulation at the cellular level, and suggested that the faster the growth rate the greater was the DNA synthesis, and greater DNA synthesis induced higher levels of [$^{18}$F]FBPA accumulation. Some studies of cell proliferation kinetics using DNA flow cytometry, bromodeoxyuridine, and [$^3$H]Thd had demonstrated constancy in the proportion of cells in each cell cycle phase, while the total cell cycle time was prolonged in accordance with the age of transplanted tumour (Skog et al., 1990; Hessels et al., 1991). A slow progression through the cell cycle was shown to be accompanied by a reduced DNA synthesis rate (Harada & Morris, 1981; Santavenere et al., 1991). In this present study, while all three tumour models showed the same S phase proportion, the DNA synthesis rate was significantly greater in B16F10. Increased DNA synthesis has been shown to be accompanied by increased protein synthesis (Bagby et al., 1992); the increased protein synthesis rate can be considered to stimulate amino acid transport/demand. [$^{18}$F]FBPA accumulation, as well as BPA accumulation, by the tumour is considered to be dependent on the amino acid transport system (Tsuji et al., 1983; Coderre et al., 1988). The transport/demand into the cells is related to the activity of DNA synthesis; however it appears that the accumulated [$^{18}$F]FBPA does not incorporate into protein synthesis, as shown by the finding of Ishiwata et al. (1992a) that [$^{18}$F]FBPA was stable to metabolic alteration in FM3A; no radioactivity was incorporated into proteins and that 89% of radioactivity was detected as [$^{18}$F]FBPA in melanoma.

On the other hand, 11% of radioactivity was detected in the acid-insoluble fraction in melanomas 1 h after injection of [$^{18}$F]FBPA. The incorporation of [$^{18}$F]FBPA into melanin production was suggested but the mechanism remains unclear. In our previous study, we demonstrated the relationship between melanogenesis and proliferation of melanoma cells with 2-[$^{18}$F]FDOPA, a DOPA analog of a melanin synthesis substrate, and with [$^3$H]Thd (Kubota et al., 1992a). We found that melanogenesis was activated only in the non-S phase melanocytes, and was increased in tissue with higher melanin content (B16F1 > B16F10).

The accumulation patterns of [$^{18}$F]FBPA, which were obtained by the same analytic technique as that used in the 2-[$^{18}$F]FDOPA study, were different from those of 2-[$^{18}$F]-FDOPA and were primarily related to the DNA synthesis activity. Poorly pigmented but highly DNA synthesising B16F10 showed higher [$^{18}$F]FBPA accumulation than B16F1; this observation is consistent with the preliminary results of Coderre et al. (1987). However, the melanocyte-related accumulation of [$^{18}$F]FBPA in B16F1, which had less DNA synthesis activity but higher melanin content than B16F10, was the same as that in B16F10, regardless of S or non-S phase cells. It may be reasonable to consider that this melanocyte-related [$^{18}$F]FBPA accumulation, which was greater in the cells with higher melanin content, was a result of [$^{18}$F]FBPA participation in melanin production.

In conclusion, we consider that two independent mechanisms account for [$^{18}$F]FBPA accumulation in the tumour cells. The primary one is amino acid transport/demand which responds to the activity of DNA synthesis, the rate of DNA synthesis being related to the *in vivo* tumour growth rate. The [$^{18}$F]FBPA accumulated by this mechanism does not incorporate into protein synthesis. The second mechanism is melanin incorporation, which is increased with melanin content. The metabolism is unclear, but the participation in melanin production is considered. These observations regarding tracer uptake mechanisms suggest that the therapeutic efficacy of BNCT using BPA may be higher in melanomas that have high DNA synthesis activity, therefore high growth rate, and high melanin content.

We hope this study will aid in the understanding of the pharmacokinetics of $^{10}$B-BPA in melanoma. The relative concentration of this compound can be assessed non-invasively *in vivo* by PET, using a $^{18}$F-labelled tracer; [$^{18}$F]FBPA. The development of positron-labelled boron compounds and the study of their kinetics may be a new approach for making progress with BNCT.

The authors are grateful to the staff of the Cyclotron and Radioisotope Center, Tohoku University, and Dr Y. Mishima (Kobe Univ., Japan) for their cooperation, and to Dr Nobuaki Tamahashi (pathologist, Clustercore Institute of Biology, Japan) for his microscopic examination and suggestions on histological procedures. This work was supported by grants-in-aid (No. 03152018, 03454277, 04557047) from the Ministry of Education, Science, and Culture, Japan.

### References

BARTH, R.F., SOLOWAY, A.H. & FAIRCHILD, R.G. (1990). Boron neutron capture therapy of cancer. *Cancer Res.*, **50**, 1061–1070.

BAGBY, S.P., O'REILLY, M.M., KIRK, E.A., MITCHELL, L.H., STENBERG, P.E., MAKLER, M.T. & BAKKE, A.C. (1992). EGF is incomplete mitogen in porcine aortic smooth muscle cells: DNA synthesis without cell division. *Am. J. Physiol.*, **262**, C578–588.

BENNETT, D.C. (1983). Differentiation in mouse melanoma cells: initial reversibility and an on-off stochastic model. *Cell*, **34**, 445–453.

CODERRE, J.A., GLASS, J.D., FAIRCHILD, R.G., ROY, U., COHEN, S. & FAND, I. (1987). Selective targeting of boronophenylalanine to melanoma in BALB/c mice for neutron capture therapy. *Cancer Res.*, **47**, 6377–6383.

CODERRE, J.A., KALEF-EZRA, J., FAIRCHILD, R.G., MICCA, P.L., REINSTEIN, L.E. & GLASS, J.D. (1988). Boron neutron cancer therapy of melanoma. *Cancer Res.*, **48**, 6313–6316.

CORY, J.G. & WHITFORD, T.N. (1972). Ribonucleotide reductase and DNA synthesis in Ehrlich ascites tumor cells. *Cancer Res.*, **32**, 1301–1306.

HARADA, J.J. & MORRIS, D.R. (1981). Cell cycle parameters of chinese hamster ovary cells during exponential polyamine-limited growth. *Mol. Cell. Biol.*, **1**, 594–599.

HESSELS, J., KINGMA, A.W., MUSKIET, F.A.J., SARHAN, S. & SEILER, N. (1991). Growth inhibition of two solid tumors in mice, caused by polyamine depletion, is not attended by alteration in cell-cycle phase distribution. *Int. J. Cancer*, **48**, 697–703.

ICHIHASHI, M., NAKANISHI, T. & MISHIMA, Y. (1982). Specific killing effect of $^{10}$B-para-boronophenylalaine in thermal neutron capture therapy of malignant melanoma: *in vitro* radiobiological evaluation. *J. Invest. Dermatol.*, **78**, 215–218.

ISHIWATA, K., IDO, T., MEJIA, A.A., ICHIHASHI, M. & MISHIMA, Y. (1991*a*). Synthesis and radiation dosimetry of 4-borono-2-[$^{18}$F] fluoro-D,L-phenylalanine: a target compound for PET and boron neutron capture therapy. *Appl. Radiat. Isot.*, **4**, 325–328.

ISHIWATA, K., IDO, T., KAWAMURA, M., KUBOTA, K., ICHIHASHI, M. & MISHIMA, Y. (1991*b*). 4-Borono-2-[$^{18}$F]fluoro-D-L-phenylalanine as a target compound for boron neutron capture therapy: tumor imaging potential with positron emission tomography. *Nucl. Med. Biol.*, **18**, 745–751.

ISHIWATA, K., KUBOTA, K., KUBOTA, R., IWATA, R., TAKAHASHI, T. & IDO, T. (1991*c*). Selective 2-[$^{18}$F]fluorodopa uptake for melanogenesis in murine metastatic melanomas. *J. Nucl. Med.*, **32**, 95–101.

ISHIWATA, K., IDO, T., HONDA, C., KAWAMURA, M., ICHIHASHI, M. & MISHIMA, Y. (1992*a*). 4-Borono-2-[$^{18}$F]fluoro-D,L-phenylalanine: a possible tracer for melanoma diagnosis with PET. *Nucl. Med. Biol.*, **19**, 311–318.

ISHIWATA, K., SHIONO, M., KUBOTA, K., YOSHINO, K., HATAZAWA, J., IDO, T., HONDA, C., ICHIHASHI, M. & MISHIMA, Y. (1992*b*). A unique *in vivo* assessment of 4-[$^{10}$B]borono-L-phenylalanine in tumor tissues for boron neutron capture therapy of malignant melanomas using positron emission tomography and 4-borono-2-[$^{18}$F]fluoro-L-phenylalanine. *Melanoma Res.*, **2**, 171–179.

KUBOTA, K., KUBOTA, R. & MATSUZAWA, T. (1983). Dose-responsive growth inhibition by glucocorticoid and its receptors in mouse teratocarcinoma OTT6050 *in vivo*. *Cancer Res.*, **43**, 787–793.

KUBOTA, R., YAMADA, S., ISHIWATA, K., KUBOTA, K. & IDO, T. (1992*a*). Active melanogenesis in non-S phase melanocytes in B16 melanoma in vivo investigated by double-tracer microautoradiography with $^{18}$F-fluorodopa and $^{3}$H-thymidine. *Br. J. Cancer*, **66**, 614–618.

KUBOTA, R., YAMADA, S., KUBOTA, K., ISHIWATA, K., TAMAHASHI, N. & IDO, T. (1992*b*). Intratumoral distribution of $^{18}$F-fluorodeoxyglucose *in vivo*: high accumulation in macrophages and granulation tissues studied by microautoradiography. *J. Nucl. Med.*, **33**, 1972–1980.

MISHIMA, Y., HONDA, C., ICHIHASHI, M., OBARA, H., HIRATSUKA, J., FUKUDA, H., KARASHIMA, H., KOBAYASHI, T., KANDA, K. & YOSHINO, K. (1989*a*). Treatment of malignant melanoma by single thermal neutron capture therapy with melanoma-seeking $^{10}$B-compound. *Lancet*, **2**, 388–389.

MISHIMA, Y., ICHIHASHI, M., TSUJI, M., HATTA, S., UEDA, M., HONDA, C. & SUZUKI, T. (1989*b*). Treatment of malignant melanoma by selective thermal neutron capture therapy using melanoma-seeking compound. *J. Invest. Dermatol.*, **92**, 321S–325S.

SANTAVENERE, E., CATALDI, A., RANA, R., VITALE, M., LISIO, R., DI DOMENICANTONIO, L., ZAMAI, L., TRUBIANI, O. & MISCIA, S. (1991). Nuclear metabolic changes induced by tumor necrosis factor in Daudi lymphoma cells; a multi parametric analysis. *Cell Biol. Int. Rep.*, **15**, 1235–1242.

SKOG, S., HE, Q. & TRIBUKAIT, B. (1990). Lack of correlation between thymidine kinase activity and changes of DNA synthesis with tumour age: an *in vivo* study in Ehrlich ascites tumour. *Cell Tissue Kinet.*, **23**, 603–617.

TSUJI, M., ICHIHASHI, M. & MISHIMA, Y. (1983). $^{10}B_1$-paraboronophenylalanine-HCl to malignant melanoma: for thermal neutron capture therapy. *Nippikaishi*, **93**, 773–778 (in Japanese).

TSUYA, A. & SHIGEMATSU, A. (1978). Microautoradiography. In *Autoradiography*, Mizuhara, V. (ed.) pp. 72–129, Ishiyaku Shuppan Co., Tokyo.

YAMADA, S., KUBOTA, R., KUBOTA, K., ISHIWATA, K. & IDO, T. (1990). Localization of [$^{18}$F]fluorodeoxyglucose in mouse brain neurons with micro-autoradiography. *Neurosci. Lett.*, **120**, 191–193.